# Factors Affecting Tablet Disintegration by Immersion in Food Thickener Solution

**DOI:** 10.7759/cureus.97179

**Published:** 2025-11-18

**Authors:** Shinya Sugiura, Megumi Nakamura, Miku Hashimoto, Chie Matsubara, Satoshi Yoshida, Yoshihiko Hirata, Michiko Obara

**Affiliations:** 1 Pharmaceutical Sciences, Graduate School of Pharmaceutical Sciences, Teikyo Heisei University, Tokyo, JPN; 2 Saraya Research Institute, Saraya Co. Ltd, Osaka, JPN; 3 Pharmaceutical Sciences, Faculty of Pharmaceutical Sciences, Teikyo Heisei University, Tokyo, JPN

**Keywords:** disintegration, food thickener, immersion, impaired swallowing, tablet

## Abstract

Food thickeners are often used to prevent aspiration in people with impaired swallowing because they can be added to foods and beverages to increase their viscosity. Although they are also used in medication administration, they affect tablet disintegration. However, the factors that delay disintegration remain unclear. In the present study, three antihypertensive agents with different characteristics were immersed in thickened solutions and subjected to disintegration testing to evaluate the effects of the type of food thickener (xanthan gum vs. starch), presence or absence of a disintegrant, and immersion time (0, 1, and 30 min) on tablet disintegration time under Japanese Pharmacopoeia conditions. Linear regression analysis was performed on the results. When Blopress^®^ tablets containing disintegrants were immersed in a xanthan gum-based thickener solution, the disintegration time was prolonged by approximately 80-300 s, and linear regression analysis showed that this prolongation was significant. Meanwhile, the disintegration time of Norvasc® tablet (5 mg), which also contains a disintegrant, was 42 s without immersion and extended to 187 s after immersion in a thickening solution of xanthan gum for 30 min. Furthermore, the starch-based thickener solution did not affect tablet disintegration regardless of the presence or absence of disintegrants.

## Introduction

Food thickeners are added to foods and beverages to prevent aspiration and choking in individuals with impaired swallowing function [1,2]. Food thickeners are also used for medication administration. However, xanthan gum-based thickeners reportedly affect tablet disintegration [3,4]. Several people consume tablets with a thickened solution [4]; therefore, countermeasures for taking tablets with a thickened solution have recently been studied.

Some studies have investigated factors affecting tablet disintegration in thickened solutions. For example, the effect of a thickened solution on tablet disintegration differs depending on the tablet specifications and manufacturer, even if the active ingredients are the same [5,6]. We previously performed disintegration testing in thickened solution by immersing three types of amlodipine besilate tablets (Norvasc® tablet) with different specifications and ingredient ratios and showed that the effect on disintegration differed among them [7]. However, we found no correlation between tablet specifications, active ingredient content, or disintegration time. Other factors may also affect tablet disintegration, and further testing of different tablets is necessary to evaluate them. Therefore, it is important to investigate the factors affecting the disintegration of tablets immersed in thickened solutions when considering countermeasures for medication administration. In evaluating what factors affect disintegration time other than tablet specifications and active ingredient content, we performed disintegration tests of Norvasc® tablet, as well as those tablets with multiple specifications (Maintate® tablet, Blopress® tablet), immersed in thickened solutions, and evaluated the factors affecting their disintegration using linear regression analysis. Essentially, we attempted to quantify the effects of the type of food thickener (xanthan gum vs. starch), presence or absence of a disintegrant (carmellose calcium and sodium carboxymethyl starch), and immersion time. Based on previous reports [8], we predicted that the disintegration time of tablets containing a disintegrant would be prolonged upon immersion in thickened solutions, regardless of the immersion time.

## Materials and methods

Sample

The tablets studied herein (Norvasc®, Meintate®, and Blopress® tablets) are listed in Table 1. Detailed information on each is provided in Table 2.

**Table 1:** Medications

Generic name	Product name	Company	Lot
Amlodipine	Norvasc^®^ tablet 2.5 mg	Viatris Pharma Ltd.	HM8227
	Norvasc^®^ tablet 5 mg		HK4622
	Norvasc^® ^tablet 10 mg		HM8077
Bisoprolol fumarate	Maintate^® ^tablet 0.625 mg	Tanabe Mitsubishi Pharm Ltd.	G673A
	Maintate^®^ tablet 2.5 mg		H502A
	Maintate^®^ tablet 5 mg		H149A
Candesartan cilexetil	Blopress^®^ tablet 2 mg	Teva Takeda Pharma Ltd.	JG031
	Blopress^®^ tablet 4 mg		JJ011
	Blopress^®^ tablet 8 mg		JL011
	Blopress^®^ tablet 12 mg		JE021

**Table 2:** Specifications of tablets used *Norvasc^®^ tablet: actual measurement (n = 5); Maintate^®^ tablet and Blopress^®^ tablet: listed in attached document

Product name	Percentage of active ingredient	Weight* (mg)	Shape	Scored
Norvasc^®^ tablet 2.5 mg	2.4%	104.5 ± 0.4	Round	No
Norvasc^®^ tablet 5 mg	2.4%	207.2 ± 0.7	Round	Yes
Norvasc^® ^tablet 10 mg	3.9%	259.1 ± 1.4	Round	Yes
Maintate^® ^tablet 0.625 mg	1.0%	60	Oval	Yes
Maintate^®^ tablet 2.5 mg	2.1%	120	Round	Yes
Maintate^®^ tablet 5 mg	3.6%	140	Round	Yes
Blopress^®^ tablet 2 mg	1.5%	130	Round	No
Blopress^®^ tablet 4 mg	3.1%	130	Round	Yes
Blopress^®^ tablet 8 mg	6.2%	130	Round	Yes
Blopress^®^ tablet 12 mg	9.2%	130	Round	Yes

We used Toromi Meijin Multi-Quick (Xa) (Saraya Co., Ltd., Osaka, Japan) as the xanthan gum-based thickener. Although various xanthan gum-based thickeners are commercially available, we selected a product composed only of xanthan gum and dextrin to minimize the influence of other additives. Furthermore, we used Toromerin Granules (St) (Nutri Co., Ltd., Mie, Japan) containing modified starch and dextrin as raw materials for starch-based thickeners.

Preparation of food thickener solutions

The thickened solutions were prepared by adding 2.0 g of Xa or 3.12 g of St to 80 mL of distilled water at 20°C and mixing with a spoon for 30 s. These concentrations corresponded to the category of extremely thick viscous products (300-500 mPa·s) as defined by the Japanese Society of Dysphagia Rehabilitation 2021 classification for swallowing-adjusted foods. Viscosity was measured using a rheometer (MCR302; Anton Paar Japan Co., Ltd., Tokyo, Japan) equipped with a cone-plate geometry, with a diameter of 50 mm. The measurement gap was 0.1 mm. The shear rate was kept constant at 50 s^-1^, and viscosity measurements were taken 1 min later. The sample temperature was 20.0 ± 0.5°C. The measured viscosities of Xa and St solutions (St-Sol) were 420.0 ± 17.2 and 424.7 ± 20.0 mPa·s, respectively.

Disintegration test

The tablets listed in Table 1 were immersed in the thickened solution for 1 or 30 min and then scooped with a spoon. The tablets were then placed in test baskets and immediately subjected to disintegration testing. Non-immersed tablets served as controls. Disintegration tests were performed on one tablet at a time using a disintegration tester (COLLAPSE DEGREE TESTEHM-1E No. 14877; Miyamoto Riken Kogyo Co., Ltd., Osaka, Japan), following the method described in the 18th edition of the Japanese Pharmacopoeia. The test medium was distilled water maintained at 37 ± 2°C; no auxiliary disk was used. Each tablet was tested five times.

Statistical analysis

Data are presented as mean ± standard error. Linear regression analysis (forced entry method) was performed with disintegration time as the dependent variable and the presence of a disintegrant (sodium carboxymethyl starch (SCS) or carmellose calcium (CC)) (presence = 1, absence = 0) and immersion in each thickened solution (immersion = 1, non-immersion = 0) as independent variables. To examine the association between immersion in a thickened solution and the disintegrant, an interaction term was introduced into the linear regression model. The suitability of the correlation model was evaluated using an adjusted coefficient of determination (R^2^). Statistical analysis software R version 4.4.1 (R Foundation, Vienna, Austria) was used for this analysis.

## Results

Change in disintegration time due to immersion in thickened solution

The mean disintegration times of the tablets shown in Table 1 when immersed in the thickened solution for 1 or 30 min and not immersed are shown in Table 3.

**Table 3:** Tablet disintegration time after immersion in food thickener solutions for 1 versus 30 min St, starch-based thickener; Xa, xanthan gum-based thickener Values are shown as mean ± standard error.

		Non-immersed	Xa 1 min	Xa 30 min	St 1 min	St 30 min
Norvasc^®^ tablet	2.5 mg	75.6 ± 4.2	47.4 ± 5.8	84.8 ± 30.8	42.6 ± 3.1	< 10
	5 mg	42.4 ± 6.1	30.4 ± 0.7	187.4 ± 27.8	18.8 ± 0.4	< 10
	10 mg	58.2 ± 3.2	45.0 ± 3.3	51.4 ± 7.0	36.2 ± 0.6	< 10
Maintate^®^ tablet	0.625 mg	275.2 ± 4.7	279.4 ± 6.7	217.6 ± 3.3	272.8 ± 11.1	220.0 ± 3.0
	2.5 mg	321.6 ± 5.0	330.2 ± 3.0	276.6 ± 4.0	315.0 ± 1.6	279.4 ± 2.7
	5 mg	311.4 ± 1.7	300.0 ± 1.8	265.8 ± 3.5	301.6 ± 1.7	266.2 ± 2.7
Blopress^®^ tablet	2 mg	487.6 ± 8.1	794.0 ± 24.1	644.4 ± 19.9	481.4 ± 12.6	477.4 ± 6.8
	4 mg	490.4 ± 10.3	755.8 ± 34.7	569.4 ± 17.8	492.8 ± 11.9	422.2 ± 18.2
	8 mg	466.2 ± 4.1	730.0 ± 31.2	606.8 ± 38.4	467.8 ± 12.8	402.2 ± 12.5
	12 mg	499.8 ± 12.5	787.4 ± 30.4	655.2 ± 14.4	534.2 ± 8.7	467.4 ± 8.7

The mean disintegration time of Norvasc® tablet immersed in Xa solution (Xa-Sol) for 1 min was >10 s shorter than that of the tablets not immersed in the thickened solution. However, the mean disintegration time of Norvasc® tablet immersed for 30 min was prolonged compared to that of the non-immersed tablet. However, in the case of Maintate® tablet, the difference in mean disintegration time between the immersed and non-immersed tablets was small at 1 min of immersion. However, after 30 min of immersion, the mean disintegration time for all tablets decreased by approximately 50 s. In the case of Blopress® tablet, the mean disintegration time was prolonged regardless of immersion time, but was shorter when immersed for 30 versus 1 min.

When immersed in St-Sol, the mean disintegration time of all tablets was not prolonged; rather, it tended to decrease.

Investigation of factors affecting disintegration time using linear regression analysis

The disintegration time of only the Maintate® tablet was not prolonged when immersed in Xa-Sol (Table 3). One difference between the other two tablets was that Maintate® tablet did not contain a disintegrant (Table 4). The disintegration time of the tablet was not prolonged when immersed in St-Sol. Based on these results, a linear regression analysis was performed focusing on the presence of disintegrants and the thickened solution type (Table 5). Significant probabilities and partial regression coefficients (B) suggested that the SCS (disintegrant in Norvasc® tablet) was responsible for the shorter tablet disintegration time (B = -244.00, p < 0.001), whereas the CC (disintegrant in Blopress® tablet) was responsible for the longer disintegration time (B = 183.27, p < 0.001). The disintegration time of the tablets varied significantly in the presence of disintegrants, regardless of immersion in thickened solutions. However, immersion in the thickened solution was not a significant variable (immersion in Xa-Sol, p = 0.1454; immersion in St-Sol, p = 0.1096). Moreover, the interaction between immersion in Xa-Sol and CC was significant (B = 231.34, p < 0.001), whereas that between immersion in Xa-Sol and SCS was not significant (p = 0.0915). The interaction term between immersion in St-Sol and SCS or CC was not significant (p = 0.6825 and 0.5118, respectively), meaning that the disintegration time of the tablet containing CC (Blopress® tablet) was prolonged by immersion in Xa-Sol, whereas immersion in St-Sol had no effect.

**Table 4:** Tablet composition The purpose of the additives is our prediction. *Formulated in Blopress^®^ tablet 8 and 12 mg.

	Agent	Norvasc^®^ tablet	Maintate^®^ tablet	Blopress^®^ tablet
Disintegrants	Sodium carboxymethyl starch (SCS)	●	–	–
	Carmellose calcium (CC)	–	–	●
Binders, excipients	Cellulose	●	–	–
	Anhydrous dibasic calcium phosphate	●	–	–
	D-mannitol	–	●	–
	Corn starch	–	–	●
	Hydroxypropylcellulose	–	–	●
	Lactose monohydrate	–	–	●
Coating agent	Hypromellose	●	–	–
Lubricants	Magnesium stearate	●	●	●
	Tark	●	–	–
Others	Carnauba wax	●	–	–
	Macro goal 6000	–	●	●
	Titanium oxide	●	–	–
	Yellow no. 5	–	–	●*

**Table 5:** Effect of disintegrant on disintegration time (linear regression analysis) Adjusted R^2^: 0.9478

	Estimates (B)	p value
(Intercept)	302.73	< 0.001
Immersion in Xa-sol	-24.47	0.1454
Immersion in St-sol	-26.90	0.1096
Sodium carboxymethyl starch (SCS)	-244.00	< 0.001
Carmellose calcium (CC)	183.27	< 0.001
Immersion in Xa-sol × SCS	40.13	0.0915
Immersion in Xa-sol × CC	231.34	< 0.001
Immersion in St-sol × SCS	-15.57	0.5118
Immersion in St-sol × CC	9.08	0.6825

## Discussion

The proportion of elderly people in Japan, and consequently, that of elderly people with age-related impaired swallowing function, is increasing annually [9]. Food thickeners are widely used to prevent the aspiration of food and drinks, while thickened water or beverages are often used for medication administration, among people with impaired swallowing function [4]. Furthermore, since food thickeners are classified as “food,” they can be used safely by middle-aged and older individuals who are gradually experiencing a decline in swallowing function [10]; thus, the demand for thickening agents is expected to increase. However, individuals may continue to take medicines without knowing that food thickeners may cause problems with disintegration and absorption. In this study, we tested tablets that are often taken with thickened solutions to identify this problem in medical and nursing care facilities. Specifically, the effect of thickened solutions on the disintegration time was evaluated using representative medicines frequently prescribed for older adults, that is, antihypertensive drugs, as the prevalence of hypertension is high among the elderly, and hence, the most commonly prescribed drugs for people aged ≥60 years [11,12].

Factors affecting tablet disintegration based on the disintegration test results of Xa-Sol

Of the tablets evaluated in this study, the disintegration time of some Norvasc® tablets and Blopress® tablets was prolonged by immersion in Xa-Sol, while that of Maintate® tablets was not (Table 3). One of the differences was that the Maintate® tablets do not contain disintegrants (Table 4). Disintegrants are excipients used to disintegrate and disperse drugs into individual particles and facilitate their absorption after oral administration [13]. Sugiura et al. [8]reported that when model tablets containing various disintegrants were immersed in a xanthan gum solution, the disintegration time was prolonged, even when immersed for only 1 min. Therefore, disintegrants are considered to be involved in prolonging the disintegration times.

Factors affecting tablet disintegration based on the disintegration test results of St-Sol

In this study, tests were performed using a starch-based thickener in the same manner as the xanthan gum-based thickener. There have been several reports on the effect of immersion in xanthan gum-containing thickened solutions on tablet disintegration and dissolution times; however, there have been few reports on starch-containing thickeners. For the tablets used in this study, immersion in St-Sol had little effect on the disintegration time but tended to shorten it (Table 3). Unlike xanthan gum, starch is considered to interact little with the disintegrants.

Factors affecting tablet disintegration based on linear regression analysis

Linear regression analysis was performed using the interaction term between immersion in the thickened solutions and the presence of disintegrants (food thickener × SCS or CC). Immersion in Xa-Sol and the interaction term between immersion in Xa-Sol and CC (the disintegrant in Blopress® tablet) were selected as significant variables, whereas, contrary to expectations, the interaction term between immersion in Xa-Sol and SCS (the disintegrant in Norvasc®tablet) was not (p = 0.0915; Table 5). The reason for this discrepancy could be due to the difference in the type of disintegrant used. A test of model tablets revealed that the degree of disintegration (prolonged disintegration time) by immersion in Xa solution for 1 min differed depending on the type of disintegrant [8]. However, while the disintegration time was prolonged for all model tablets, the disintegration time was shortened when Norvasc® tablet was immersed for 1 min in this study (Table 3).

Therefore, the interaction term between immersion in Xa-Sol and SCS was not selected as a significant variable for other reasons. One reason could be that Norvasc® tablet is film-coated. The disintegration time of film-coated tablets is reportedly suppressed when immersed in the Xa solution for 1 min [14]. However, in this study, the disintegration time tended to be prolonged when immersed in the Xa solution for 30 min (Table 3). For a short immersion time of approximately 1 min, the Xa solution may not come in direct contact with the tablet components (disintegrant). However, the prolonged disintegration time when immersed in Xa solution for 30 min suggests that an interaction may have occurred between the Xa solution and tablet components. Elucidating this complex phenomenon requires surface science analysis, which is a subject for future research. Therefore, we speculate that the interaction term between SCS and xanthan gum may be significant if tablets contain SCS but are not film-coated.

 Furthermore, in the regression model used in this study, a binary variable (presence or absence) was used for disintegrants. Because tablet formulations are generally not disclosed, we could not evaluate whether the disintegrant content affects the disintegration time. Given that disintegrant content could be a potential confounding factor, it is desirable to conduct future evaluations using model tablets.

The limitation of the study

This study has several limitations. First, the evaluation of the factors affecting disintegration time was limited. This study suggests that the coexistence of xanthan gum and disintegrants affects the disintegration time. However, other factors may influence disintegration time. For example, the tablet shape and score lines are possible factors; however, they could not be evaluated in this study because of the small sample size. Second, we tested food thickeners composed of only xanthan gum, starch, and dextrin. Some food thickeners contain guar gum or carrageenan as thickening agents. The disintegration patterns of several types of MgO tablets reportedly differ between xanthan gum-based and guar gum-based food thickeners [15]. Therefore, the results of this study may not be applicable to all food thickeners. Third, the immersion time of tablets in the thickened solution was limited. In this study, the immersion time was limited to either 1 or 30 min, and no other time or conditions were verified. Particularly in clinical practice, various usage situations are conceivable, such as immersion in a thickened solution immediately before medication administration or letting it sit for a while after immersion. Fourth, the number of tablets used in this study was limited. Because the study was conducted on only three types of antihypertensive drugs, we need to investigate whether similar results can be obtained with other drug classes, such as antibiotics, gastrointestinal drugs, and sleeping pills. However, the application of this linear regression analysis is limited, and the results should be interpreted with caution. Fifth, the disintegration test conditions may differ from those used in clinical practice. Although this study evaluated the disintegration time according to the method of the 18th edition of the Japanese Pharmacopoeia, conditions such as moisture content, temperature, agitation state (peristalsis), and gastrointestinal retention time differed from those of the actual medication administration. Furthermore, in vitro conditions may differ from the actual disintegration behavior in the human body because saliva and gastric acid are present during swallowing and absorption. Therefore, it is recommended that tests be conducted using more complex models that more accurately resemble the behavior within the human body.

This study demonstrated that different tablet types and specifications affect tablet disintegration in food thickeners. Our findings provide information on the appropriate use of food thickeners so that everyone can safely and effectively take medications containing food thickeners.

## Conclusions

The results of this study confirmed that the disintegration time of Blopress® tablets, which contain a disintegrant, was significantly prolonged when they were immersed in Xa-Sol. Although Norvasc® tablets contain a disintegrant, their disintegration time when immersed in Xa-Sol for 1 min was not prolonged. This was presumably due to the effect of the coating. Therefore, when individuals plan to take disintegration-containing tablets with a thickened solution containing xanthan gum, measures such as shortening the immersion time are recommended. Our findings also suggest that a starch-containing thickener may not significantly affect tablet disintegration time, regardless of the presence or absence of a disintegrant.
